# Establishment of a BALB/c Mouse Model for Photoaged Skin: Insights into UV‐Induced Dermatological Changes

**DOI:** 10.1111/srt.70353

**Published:** 2026-04-23

**Authors:** Cuc Bach Huynh, Ngoc Bich Vu, Trung The Van, Phuc Van Pham

**Affiliations:** ^1^ VNUHCM‐US Stem Cell Institute University of Science Ho Chi Minh City Viet Nam; ^2^ Department of Dermatology University of Medicine and Pharmacy at Ho Chi Minh City Ho Chi Minh City Viet Nam; ^3^ Viet Nam National University Ho Chi Minh City Ho Chi Minh City Viet Nam

**Keywords:** murine model, photoaging, skin aging, ultraviolet exposure

## Abstract

**Background:**

Photoaging, primarily induced by prolonged ultraviolet (UV) radiation exposure, significantly alters skin structure and function, necessitating models for deeper understanding and intervention development. This study aims to establish a BALB/c mouse model for investigating photoaging, leveraging their haired skin and genetic consistency.

**Materials and Methods:**

Six‐week‐old female BALB/c mice were subjected to repeated exposure to Ultraviolet A (315–400 nm) (UVA) and Ultraviolet B (280–315 nm) (UVB) radiation at doses corresponding to one and two minimal erythemal doses (MEDs), four times weekly for 12 weeks. Skin aging was evaluated through clinical observations of wrinkles, thickness, hydration, and elasticity, as well as histopathological analyses of epidermal and dermal thickness and collagen degradation. Additionally, gene expression was conducted for collagen Types I and III, and matrix metalloproteinases (MMPs) 1, 2, and 3.

**Results:**

UV‐exposed mice exhibited significant increases in skin thickness and wrinkle formation, with concurrent decreases in hydration and elasticity compared to controls. Aging signs were more pronounced in the two MED‐group than in the one MED‐group. Histological assessment showed epidermal hyperplasia, dermal thickening, and collagen degradation. Furthermore, UV irradiation upregulated MMP‐1, MMP‐2, and MMP‐3 expression while downregulating Collagen 1 and 3, indicating molecular pathways of skin aging.

**Conclusions:**

The BALB/c mouse model effectively mimics human photoaging under chronic UV exposure using a two‐MED protocol, which corresponds to a total dose of 51.8 J/cm^2^ UVA and 5.53 J/cm^2^ UVB. This practical model, which exhibits skin features comparable to those of humans, provides a closer approximation of human skin responses for comprehensive studies on the mechanisms and interventions of photoaging.

**Abbreviations**
ANOVA: analysis of variance
BALB/c: Bagg Albino, substrain c
Col1a1, Col3a1, Mmp1b, Mmp2, Mmp3 : Specific genes coding for collagen type 1 alpha 1, collagen type 3 alpha 1, matrix metalloproteinase‐1b, matrix metalloproteinase‐2, and matrix metalloproteinase‐3, respectively.
Gapdh: glyceraldehyde 3‐phosphate dehydrogenase (used as a control gene in molecular biology experiments)
H&E: hematoxylin and eosin
LACU: Laboratory for Animal Care and Use
LSD: least significant difference
MED: minimal erythemal dose
MMP: matrix metalloproteinase
mRNA: messenger RNA
PCR: polymerase chain reaction
RT‐PCR: reverse transcription polymerase chain reaction
SKH‐1: a specific strain of hairless mice used in dermatological research
SPSS: Statistical Package for the Social Sciences
UV: ultraviolet
UVA: Ultraviolet A (315–400 nm)
UVB: Ultraviolet B (280–315 nm)

## 1 Introduction

Photoaging represents a critical concern in dermatology, characterized by premature skin aging resulting from chronic exposure to ultraviolet (UV) radiation [1]. This process is distinct from chronological aging and is marked by significant changes in the appearance and structural integrity of the skin, largely attributed to the cumulative effects of UV from the sun [2]. Clinically, photoaged skin is recognizable by its coarse wrinkles, loss of elasticity, dryness, and uneven pigmentation, which are not only cosmetic issues but also potential precursors to skin malignancies [1, 3]. At the histological level, photoaging is characterized by epidermal thickening, increased dermal collagen degradation, and the accumulation of abnormal elastin‐containing materials, known as solar elastosis [1, 3]. Given the impact of premature photoaging on both the aesthetic and physiological functions of the skin, research in this area, particularly the development of effective models for evaluating interventions, continues to attract increasing scientific interest.

The pathogenesis of photoaging involves complex molecular mechanisms driven by UV‐induced DNA damage, oxidative stress, inflammation, immune suppression, and the upregulation of matrix metalloproteinases (MMPs), leading to the accelerated breakdown of collagen and other extracellular matrix components [4]. These processes underscore the intricate interplay between UV exposure and skin physiology, necessitating in‐depth studies on disease models to elucidate the underlying mechanisms and identify potential targets for prevention and treatment. Nevertheless, existing models of photoaging still exhibit limitations that warrant further investigation and refinement.

Traditionally, the investigation of photoaging has been hampered by the ethical and logistical challenges of conducting long‐term studies on human subjects exposed to UV [5]. Animal models, particularly rodents, have emerged as crucial alternatives, enabling controlled studies on the effects of UV on skin aging. The hairless mouse model, especially a specific strain of hairless mice used in dermatological research (SKH‐1) strain, has been extensively used in photoaging research due to its lack of fur, facilitating direct UV exposure without the need for depilation [6]. These models have contributed valuable insights into the dermatological and molecular alterations associated with photoaging, including the validation of photoaging markers and the testing of photoprotective compounds.

However, the hairless mouse model presents several limitations that may affect the translatability of findings to human skin. The absence of hair follicles in these mice eliminates an important aspect of human skin physiology, potentially influencing the response of the skin to UV [6, 7]. Moreover, the genetic variability and outbred nature of SKH‐1 mice introduce additional variables that can complicate the interpretation of experimental results [6, 7].

In response to these challenges, this study proposes the usage of the BALB/c mouse, an inbred strain with haired skin, as a novel model for photoaging research. The BALB/c mouse strain stands out as one of the most extensively utilized inbred strains in biomedical research. The BALB/c mouse offers several advantages, including genetic uniformity, which minimizes experimental variability and enhances study reproducibility [8, 9]. Notably, BALB/c mice serve as a standard model for investigating inflammatory cytokines, reactive oxygen, and molecular signaling pathways such as NF‐κB and MAPK, thereby enabling detailed analysis of the mechanisms underlying Ultraviolet B (280–315 nm) (UVB)‐induced skin aging [10, 11, 12]. Furthermore, the presence of hair and other skin appendages in BALB/c provides a closer approximation to human skin [6, 7]. BALB/c mice could serve as a practical and alternative model for photoaging studies alongside SKH‐1 mice.

Although SKH‐1 mice possess skin structures more comparable to human skin and do not require hair removal [6], they are significantly more expensive and less accessible than BALB/c mice. Currently, photoaging models using BALB/c mice are less common than those using SKH‐1 mice, and the skin responses of BALB/c mice to UV‐induced aging remain poorly understood, with limited comparative studies available.

By subjecting BALB/c mice to chronic UV irradiation, this study aims to replicate the human condition of photoaging more accurately, offering new perspectives on the molecular and cellular processes driving skin aging under the influence of UV. The goal is to explore the suitability of BALB/c mice as a model for UV‐induced skin aging and to contribute an additional, accessible option for photoaging research in animal models.

This comprehensive approach seeks to fill the gap in knowledge about photoaging and explore the efficacy of potential interventions in a model that more closely mimics human skin. Through detailed investigations of skin wrinkles and barrier function, as well as epidermal and dermal thickness, collagen density, and the expression of collagen and MMPs in BALB/c mice, the current study attempts to shed light on the complex dynamics of photoaging, paving the way for novel strategies to prevent and treat UV‐induced skin damage.

## 2 Materials and Methods

### 2.1 Animals and Ethical Approval

This study utilized 6‐week‐old female BALB/c mice obtained from the Laboratory for Animal Care and Use (LACU) at the Stem Cell Institute, University of Science, Vietnam National University, Ho Chi Minh City, Vietnam. Animals were allowed to acclimatize for 7 days prior to the experimentation under standardized laboratory conditions (temperature 22 ± 2°C, relative humidity approximately 55%, with a 12‐hour light/dark cycle). Body weight was recorded twice weekly during the study period. All procedures involving animals were conducted in accordance with LACU regulations and were approved by the Institutional Animal Care and Use Committee of the Stem Cell Institute (approval no. 210103/SCI‐AEC; January 20, 2021) [13].

### 2.2 UV‐induced Aging Mouse Model

A UV lamp (Exo Terra Reptile UVB150, 18W‐60 cm, PT2396, Exo Terra, Canada) emitting both Ultraviolet A (315–400 nm) (UVA) and UVB light (290–370 nm) was used. The energy output of the lamp was calibrated using an Extech UV‐AB light meter (model UV505, Wilsonville, USA) and a Santacary XAR‐UV UVB light meter (Guangdong, China), positioned 30 cm from the animals.

Fifty‐four mice were randomly divided into three groups: control (no UV exposure), UV1 (exposed to 1 minimal erythemal dose [MED] of UV), and UV2 (exposed to 2 MED of UV), with 18 mice per group. The dorsal fur of the mice was shaved bi‐weekly to ensure direct UV exposure. The skin was shaved without anesthesia. The mouse was gently placed on a soft cloth, and another soft cloth was used to cover its head. Warm water was applied to moisten the area to be shaved, and the hair was carefully shaved in the direction of growth, avoiding skin injury and minimizing stress to the animal. Additionally, the shaving was performed one day before UV exposure to allow sufficient time for the skin to recover, thereby minimizing any confounding effects of UV irradiation on sensitized skin. Mice were placed in individual self‐designed exposure chambers (3 × 6 cm) to limit movement without the need for anesthesia, exposing their dorsal surfaces to UV light. The temperature of the mice's skin was maintained at approximately 38°C to minimize the effect of heat on the skin.

Irradiation doses were determined through preliminary experiments to establish the MED. To determine MED, the dorsal skin of the mice was exposed to varying doses of UV light, and erythemal formation was evaluated within 24 h without bullous formation [14]. Mice were exposed to UV for 10, 20, and 30 min, and 1 MED was confirmed as 30 min‐UV, which is equivalent to 540 mJ/cm^2^ UVA and 57.6 mJ/cm^2^ UVB. Following this, a pilot study was conducted on 12 BALB/c mice to determine the appropriate UV dose for inducing photoaging. Before UV exposure, the mice exhibited pale pink, smooth skin. After irradiation with a dose equivalent to 1 MED, mild erythema appeared and resolved spontaneously within 4 h. At 2 MED, the erythema was more pronounced and resolved within 12 h. However, exposure to 3 MED led to edema, which progressed to erosion and crust formation after 48 h. Based on these observations, 1 MED (540 mJ/cm^2^ UVA and 57.6 mJ/cm^2^ UVB) and 2 MED (1080 mJ/cm^2^ UVA and 115.2 mJ/cm^2^ UVB) doses were chosen for further experiments as the UV1 and UV2 groups. UV exposure was administered for 30 min per day for the UV1 group and 60 min per day for the UV2 group, four consecutive days weekly for 12 weeks. The total accumulative doses were 25.9 J/cm^2^ UVA and 2.76 J/cm^2^ UVB for the UV1 group, and 51.8 J/cm^2^ UVA and 5.53 J/cm^2^ UVB for the UV2 group. Post‐exposure, the mice were sacrificed, and skin tissues were collected for analysis. The mouse was euthanized using CO_2_ inhalation. The animal was placed in a chamber, and CO_2_ was pumped in for 10 seconds. Once the mouse was no longer moving, dissection procedures were performed.

#### 2.2.1 Body Weight Changes

Body weights were measured every 2–4 weeks from Week 1 to Week 12. All values are presented as mean ± standard deviation (SD). Three technical replications were performed for each mouse at each time point.

#### 2.2.2 Wrinkle Formation Measurement

The degree of UV‐AB‐induced skin aging was assessed by evaluating wrinkle formation, which was conducted by two independent observers every 2–4 weeks. Shaved mice were irradiated with UV‐AB on the dorsal region for 12 weeks to induce photoaging. The irradiated dorsal area was photographed using a light microscope (magnification, x100). Wrinkle formation was assessed according to the scoring system established by Bissett et al. [15], which includes four levels: Grade 0 (no coarse wrinkles), Grade 1 (few shallow coarse wrinkles), Grade 2 (some coarse wrinkles), and Grade 3 (several deep coarse wrinkles). Three technical replications were performed for each mouse at each time point.

#### 2.2.3 Skin Hydration Measurement

Mice were maintained in a climate‐controlled room (22°C and 55% humidity) for 30 min before measurement. The dorsal skin of each mouse was examined using a Corneometer CM825 probe (Courage + Khazaka electronic GmbH, Köln, Germany), which was placed in close contact with the surface of the dorsal skin and lightly pressed to record the skin moisture content. Each mouse was measured three times every two weeks [13].

#### 2.2.4 Pinch Test to Evaluate Skin Elasticity

The dorsal skin of each mouse was carefully pinched between the thumb and index finger and elevated to the maximum extent without raising the animal. Subsequently, the pinched skin was released, and the time (in seconds) until the skin returned to its normal condition was recorded [16]. Three technical replications were performed for each mouse at each time point.

#### 2.2.5 Skinfold Thickness Measurement

The thickness of the skin was measured using a digital caliper (Mitutoyo, Japan). The back skin of shaved mice was manually pinched, and the micrometer caliper was applied to the skin pinch, with three technical replicate measurements each time per mouse [13].

#### 2.2.6 Histological Staining Analysis

Histological analysis was performed to determine epidermal thickness, dermal thickness, and collagen content. A total of 18 mice per group were used. Six mice were monitored longitudinally for clinical observations at Weeks 0, 4, 6, 8, and 12, as mentioned above. The remaining 12 mice were used for tissue collection for hematoxylin and eosin (H&E) and collagen analyses at Weeks 0, 4, 6, and 8 (three mice per time point). At Week 12, all six mice from the longitudinal observation subset were sacrificed for histological and molecular analyses.

In addition, three extra mice were included in each group as backup to compensate for potential sample loss. Since no animal loss occurred during the experiment, these reserve mice were further followed up and sampled at Week 20 (8 weeks after completing UV irradiation) for exploratory analysis. Each mouse was sacrificed immediately after tissue collection (see Figure S1 for a schematic diagram summarizing the experimental timeline and tissue collection schedule). The three surplus mice per group were not included in the main statistical analysis but were used for extended observation at Week 20.

For staining procedures, two skin samples were obtained from each mouse. From each sample, three H&E‐stained slides and one Masson's trichrome‐stained slide were prepared, and measurements were randomly performed at five locations per slide, providing technical replicates for analysis.

Dorsal skin tissues from each experimental group were fixed in 10% formalin (Xilong, China) for 48 h at 21°C. Hematoxylin & eosin (H&E) staining (Thermo Scientific, USA) was used to measure epidermal and dermal thickness, while Masson's trichrome staining (Abcam, USA) was employed to analyze collagen content. Photographs of each stained sample slide were captured at five randomly selected zones using a camera connected to a digital optical microscope.

Epidermis thickness was measured from the top of the stratum corneum to the bottom of the basal keratinocyte layer in five randomly selected fields from each photograph. Dermis thickness was assessed by measuring the distance between the bottom of the basement membrane to the top of the hypodermis. All images of the stained samples were analyzed using AxioVision Rel. software version 4.8 (Carl Zeiss, Thailand) for thickness calculation. Collagen fibers in Masson's trichrome‐stained images were deconvolved using ImageJ software (National Institutes of Health, USA) with the color deconvolution plugin for collagen quantity analysis [17].

### 2.3 Gene Expression Analysis

Total RNA was extracted from mouse skin tissue using the phenol‐chloroform method with the easy‐BLUE Total RNA Extraction Kit (Boca Scientific Inc., Dedham, MA, USA). Real‐time reverse transcription polymerase chain reaction (RT‐PCR) was performed using the Luna Universal qPCR Master Mix (New England Biolabs, Ipswich, MA, USA) with primer sequences listed in Table 1, following the manufacturer's protocol. The final concentration of each primer was 10 µM. The standard polymerase chain reaction (PCR) conditions were 55°C for 10 min, followed by 95°C for 1 min, and then 40 cycles of 95°C for 10 s and 60°C for 45 s. Results were determined as the relative quantity value of the target group and calculated using the 2‑ΔΔCq method [18].

**TABLE 1:** Mouse primer sequences for real‐time quantitative polymerase chain reaction.

Target gene	Sequences (5′‑3′)	Amplicon size (bp)	Reference
*COL1A1*	F: CCGAACCCCAAGGAAAAGA R: CTGTTGCCTTCGCCTCTGA	266	NM_007742.4
*COL3A1*	F: TGGTCCTCAGGGTGTAAAGG R: GTCCAGCATCACCTTTTGGT	221	NM_009930.2
*MMP1*	F: AAGGCGATATTGTGCTCTCC R: CCTCATTGTTGTCGGTCCAC	159	NM_032007.3
*MMP2*	F:CAGGGAATGAGTACTGGGTCTATT R:ACTCCAGTTAAAGGCAGCATCTAC	119	NM_008610.3
*MMP3*	F: CATCCCCTGATGTCCTCGTG R: CTTCTTCACGGTTGCAGGGA	359	NM_010809.3
*GAPDH*	F: CCCACTAACATCAAATGGGG R: ACACATTGGGGGTAGGAACA	478	NM_001411840.1

Abbreviations: F, forward; GAPDH, glyceraldehyde 3‐phosphate dehydrogenase (used as a control gene in molecular biology experiments); MMP, matrix metalloproteinase; R, reverse.

### 2.4 Statistical Analysis

Quantitative data are represented as mean ± SD or standard error of mean (SEM). Data were analyzed using Statistical Package for the Social Sciences (SPSS) version 20.0 (IBM Corp., Armonk, NY, USA) and GraphPad Prism 8. Differences between two groups were compared using Mann–Whitney test. For more than two groups, one‐way analysis of variance (ANOVA) with post‐hoc comparisons test such as Bonferroni's, least significant difference (LSD), or Tukey's tests was used as appropriate. Two‐way ANOVA was chosen for analyzing the statistical difference between data time points in groups. Results were considered statistically significant at *p* < 0.05. Graphs were generated using GraphPad Prism 8 (GraphPad Software, Inc., California, USA).

## 3 Results

### 3.1 Effects of UV Irradiation on Clinical Characteristics

#### 3.1.1 Body Weight

**FIGURE 1:**
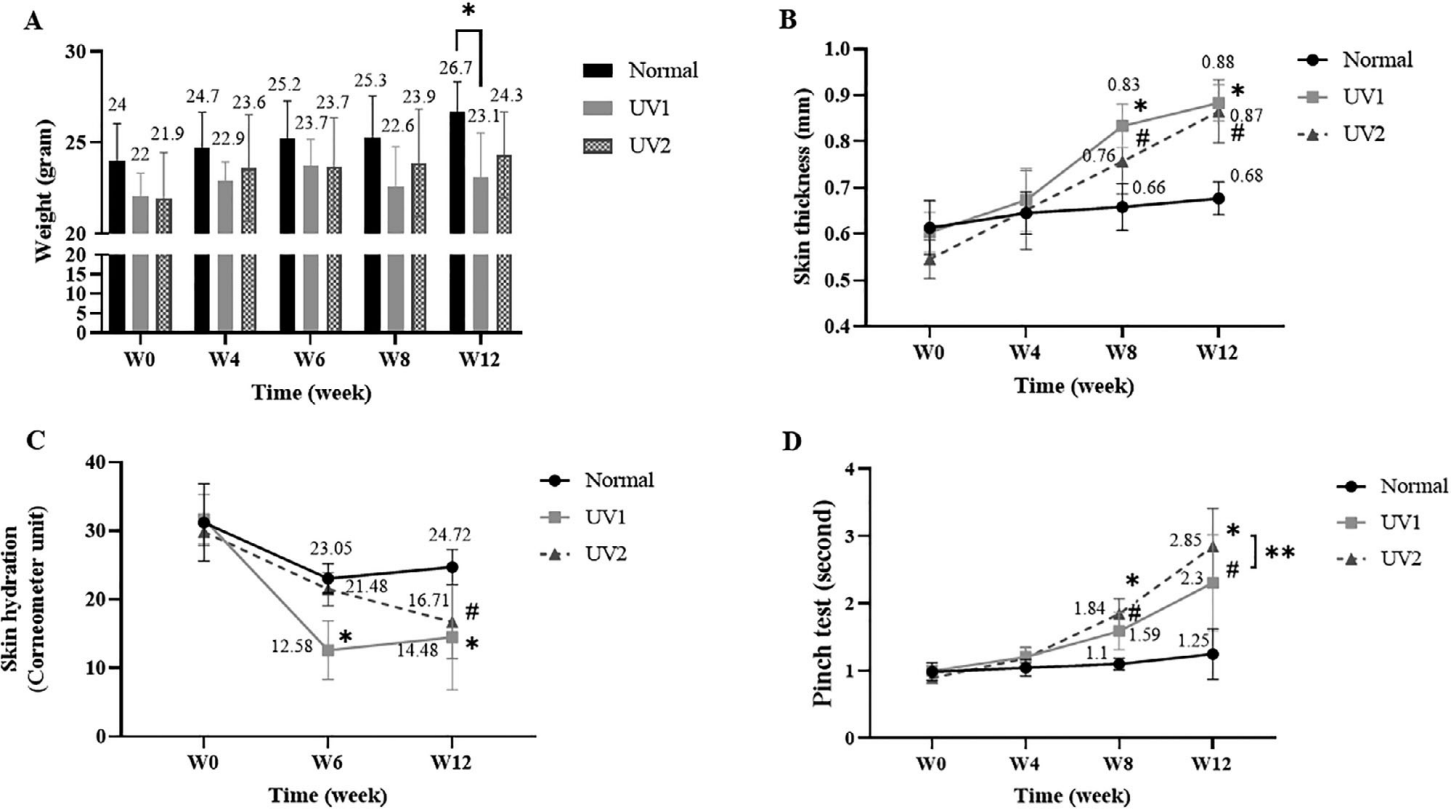
Effects of UV on clinical characteristics of mice. (A) Mouse weights change (*p < 0.05, UV1 vs. normal group); (B) Skinfold thickness was elevated in the UV‐irradiated groups (*p < 0.05, UV1 vs. normal group, ^#^ *p*<0.05, UV2 vs. normal group); (C) Skin hydration decreased in the UV‐irradiated groups compared to the normal group at Week 6 and Week 12 (*p < 0.05, UV1 vs. normal group, ^#^ *p* < 0.05, UV2 vs. normal group); (D) Skin snapback time increased in UV‐irradiated groups compared to the normal group (*p < 0.05, UV1 vs. normal group, ^#^ *p* < 0.05, UV2 vs. normal group, **p < 0.05, UV1 vs. UV2). *n* = 6 per group; UV1, UV dose was 540 mJ/cm^2^ of UVA and 57.6 mJ/cm^2^ of UVB; UV2, UV dose was 1080 mJ/cm^2^ of UVA and 115.2 mJ/cm^2^ of UVB; W: week. Data are presented as mean ± SD. Two‐way ANOVA was used for the comparisons. ANOVA, analysis of variance; SD, standard deviation; UV, ultraviolet; UVA, Ultraviolet A (315–400 nm); UVB, Ultraviolet B (280–315 nm).

The initial mean body weight of mice in the normal group was 24 ± 2.05 g, slightly higher than in the UV1 (22.07 ± 1.25 g) and UV2 (21.93 ± 2.52 g) groups, but these differences were not statistically significant (*p* > 0.05). At Weeks 4, 6, 8, and 12, both the normal and UV2 groups showed an increase in body weight over time, with no significant differences observed between these two groups. However, at Week 12, the UV1 group exhibited a significantly lower body weight (23.1 ± 2.4 g) compared to the normal group (26.7 ± 1.7 g, *p* < 0.05) (Figure 1A).

#### 3.1.2 Skinfold Thickness

Skinfold thickness increased over time in all three groups, with a dramatic increase observed in the UV‐exposed groups. At Weeks 8 and 12 of UV exposure, the skinfold thickness was significantly higher in the UV groups compared to the normal group (*p* < 0.05). Specifically, at Week 12, the skin thickness of the UV1 and UV2 groups was 0.88 ± 0.04 mm and 0.87 ± 0.07 mm, respectively, compared to the thinner skinfold observed in the normal group (0.68 ± 0.04 mm). No significant difference was revealed between two UV groups (Figure 1B).

#### 3.1.3 Skin Hydration

At Week 6, UV‐AB irradiation significantly decreased skin hydration in the UV1 group compared to the normal group, with values of 12.58 ± 4.27 and 23.05 ± 2.13, respectively (*p* < 0.001); UV2 group with skin hydration value at 21.48 ± 2.44 was also shown significant difference to UV1 group (*p* < 0.05). Until Week 12, the differences between UV1 and UV2 compared to the normal group persisted significantly, with values of 14.48 ± 7.6 and 16.71 ± 5.37 compared to 24.72 ± 2.54, respectively (*p* < 0.05), and there was no significant difference between two UV groups (Figure 1C).

#### 3.1.4 Skin Elasticity

The time for the skin to return to its original state after being stretched (the snap‐back times) in the UV‐exposed groups gradually increased from Week 0 to Week 12, whereas in the normal group, there was no change. At Week 8, the snap‐back times for the UV1 and UV2 groups were 1.590 ± 0.274 and 1.843 ± 0.224 s, respectively, significantly higher than the normal group by 1.102 ± 0.087 s (*p* < 0.05). There were no statistically significant differences between the UV1 and UV2 groups at Week 8. However, by Week 12, the UV2 group exhibited a significantly longer snap‐back time compared to the UV1 group (2.847 ± 0.562 vs. 2.305 ± 0.713 s, *p* < 0.05) and the normal group (1.248 ± 0.375 s, *p* < 0.05) (Figure 1D).

#### 3.1.5 Wrinkle Formation

**FIGURE 2:**
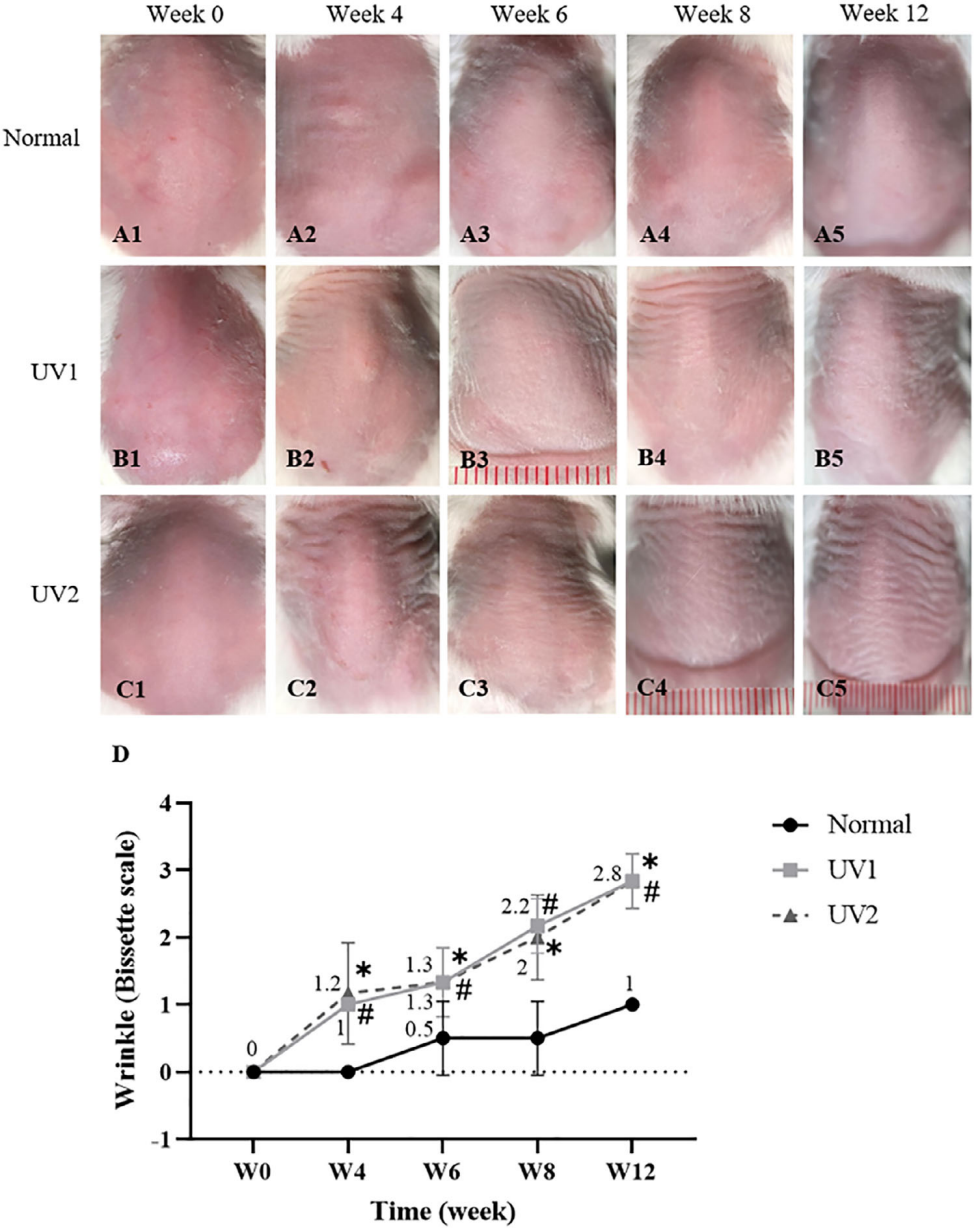
Evaluation of skin wrinkle formation in irradiated mice. (A1–A5, B1–B5, C1–C5) Surface appearance of the dorsal skin of mice; (D) Wrinkles were assessed using the Bissett wrinkle scoring system. Skin wrinkles increased in the UV‐exposed groups compared to the normal group. *n* = 6 per group; *p < 0.05, UV1 versus normal group; ^#^ *p* < 0.05, UV2 versus normal group. Data are presented as mean ± SD. Two‐way ANOVA was used for the comparisons. ANOVA, analysis of variance; SD, standard deviation; UV, ultraviolet.

At Week 0, the skin surface of mice in all three groups appeared the same: pink in color, smooth in texture, and without wrinkles (Figure 2A1, B1, C1). The skin color of the normal group remained pink with no transverse skin wrinkles throughout the observation period at Weeks 4, 6, 8, and 12 (Figure 2A2–A5). However, in the UV‐exposed groups, the skin appeared pale, and transverse skin wrinkles emerged by Week 4 (Figure 2B2, C2), covering the upper one‐third of the side back skin. The quantity and depth of these wrinkles increased progressively after 6 (Figure 2B3, C3) and 8 weeks (Figure 2B4, C4) of UV exposure. By Week 12, mice in the UV2 group exhibited more wrinkles (Figure 2C5) compared to the UV1 group (Figure 2B5), with prominent transverse deep furrows covering the entire back skin.

Furthermore, the Bissett wrinkle scores significantly increased in the UV‐exposed groups compared to the normal group by Weeks 4, 6, 8, and 12, with values of 2.83 ± 0.41 versus 1, respectively, at Week 12 (*p* < 0.05) (Figure 2D).

### 3.2 Effects of UV Irradiation on H&E Histological Alteration

**FIGURE 3:**
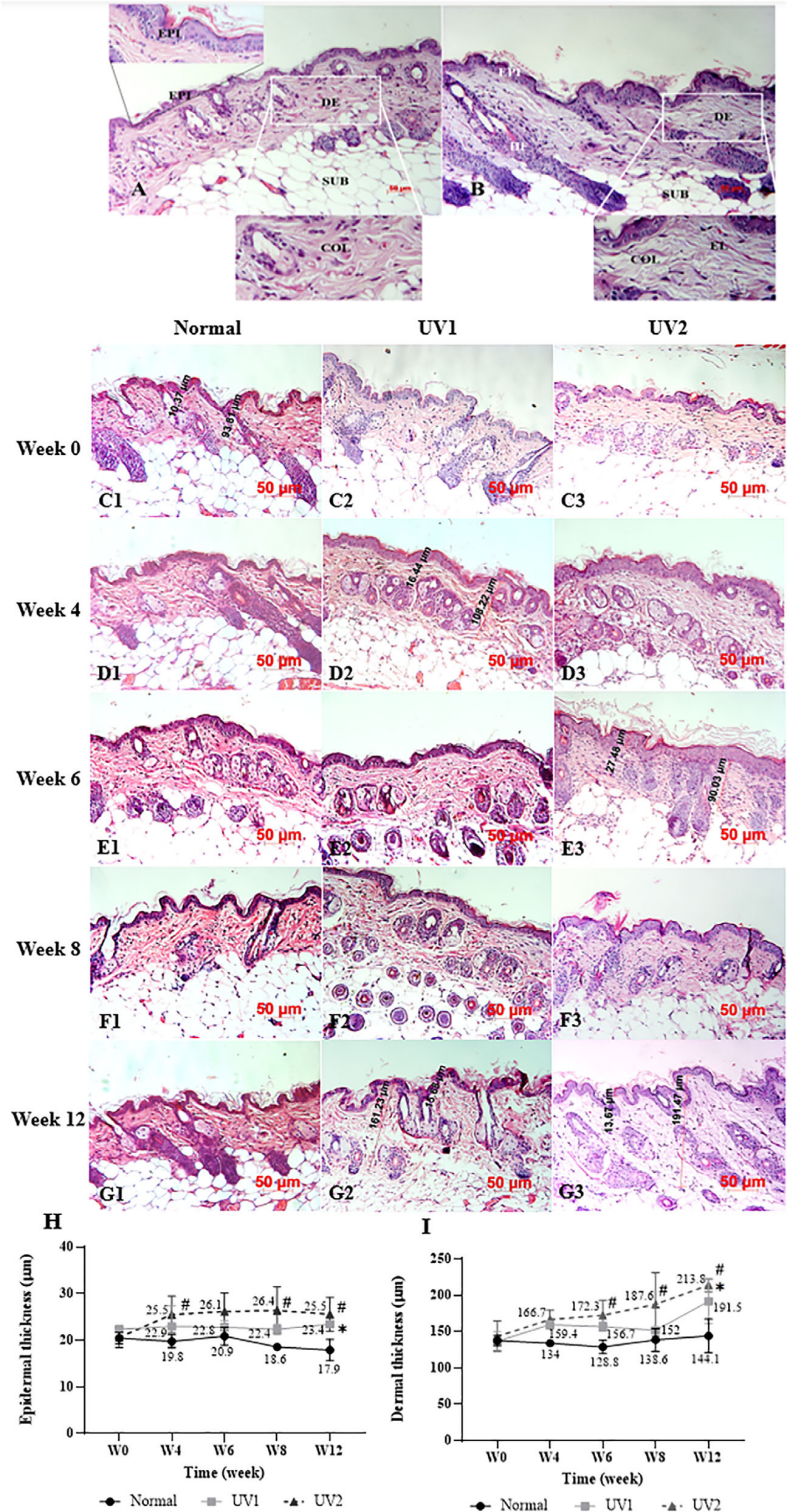
Histological alteration of mouse skin with hematoxylin and eosin (H&E) staining. (A) H&E staining of normal skin sample of mice showed three layers, epidermis (EPI), dermis (DE), and subcutaneous fat (SUB). (B) Disorganized, spared, pink‐stained collagen fibers (COL) accumulated with base‐stained elastin fibers (elastosis) (EL), no tumor lesions were observed on UV‐exposed skin with multiple hair follicles (HF) in the dermis. (C1–C3, D1–D3, E1–E3, F1–F3, G1–G3) Histological alterations stained with H&E of three mice groups at Week 0 (C1–C3), 4 (D1–D3), 6 (E1–E3), 8 (F1–F3), and 12 (G1–G3). (H) Epidermal thickness change of mice at various time points. *p < 0.05, UV1 versus normal group. ^#^ *p* < 0.05, UV2 versus normal group. (I) Dermal thickness change of mice at different time points. *p < 0.05, UV1 versus normal group. ^#^ *p* < 0.05, UV2 versus normal group. *n* = 3 per group. Data are presented as mean ± SD. Two‐way ANOVA was used for the comparisons. ANOVA, analysis of variance; SD, standard deviation; UV, ultraviolet.

In normal mice skin samples stained with H&E, the skin included three layers: epidermis, dermis, and subcutaneous fat. The epidermis was thin, with a single stratum basale, one to two layers of the stratum spinosum, a reduced stratum granulosum, no stratum lucidum, and several layers of the stratum corneum. No melanin pigment was present. The dermal‐epidermal junction appeared normal, lacking dermal papillae and epidermal ridges. The dermis presented as pink‐stained, dense, regularly arranged collagen fibers, interspersed with observable deep purple‐stained nuclei of dermal fibroblasts and multiple hair follicles (Figure 3A). After 12 weeks of UV irradiation, the pink‐stained dense collagen bundles in the UV‐exposed group were replaced by thin and sparse collagen fragments interleaved with basophilic fibrillary materials known as dermal elastosis (Figure 3B).

### 3.3 Effects of UV Irradiation on Epidermal and Dermal Thickness

Before the experiment at Week 0, skin samples were normal similar across all three groups (Figure 3C1–C3). By Weeks 4, 6, 8, and 12, the epidermis of mice in the UV1 group (Figure 3D2, E2, F2, and G2) was slightly thicker compared to the normal group (Figure 3D1, E1, F1, and G1), while in the UV2 group, the epidermis was notably thickened (Figure 3D3, E3, F3, and G3). However, by Week 12, the epidermal thickness in both UV groups had decreased compared to Weeks 4 and 6.

As shown in line graph, the epidermal thickness in the normal group slightly decreased from Week 0 to Week 12, while the dermis thickness remained consistent (Figure 3H and I). By Weeks 4, 8, and 12, the epidermal thickness significantly increased in the UV2 group compared to the normal group (*p* < 0.05). Although the epidermal thickness in UV1 group was higher than the normal group, there was no significant difference between the two groups at Weeks 4, 6, and 8 (Figure 3H). By Week 12, the epidermal thickness in the normal, UV1, and UV2 groups was measured at 17.88 ± 2.31, 23.43 ± 0.49, and 25.54 ± 3.65 µm, respectively, with significant differences noted (*p* < 0.05).

The dermal thickness in the UV2 group at Weeks 6, 8, and 12 was measured at 172.31 ± 20.78, 187.61 ± 43.48, and 213.84 ± 9.049 µm, respectively, significantly higher than in the normal group, which measured by 128.76 ± 8.96, 138.56 ± 15.71, and 144.09 ± 23.05 µm (*p* < 0.05). Similarly, the dermis in the UV1 group was thicker than that of the normal group at Week 12, measuring 191.49 ± 30.56 µm compared to 144.09 ± 23.05 µm (*p* < 0.05), although it fluctuated at Weeks 4, 6, and 8 (Figure 3I).

Taken together, these results demonstrated that the epidermal and dermal thickness of mice in the UV1 and UV2 groups increased compared to that of the normal group, with significant differences were mostly observed in the UV2 group.

### 3.4 Effects of UV Irradiation on Collagen Quantification Alteration

**FIGURE 4:**
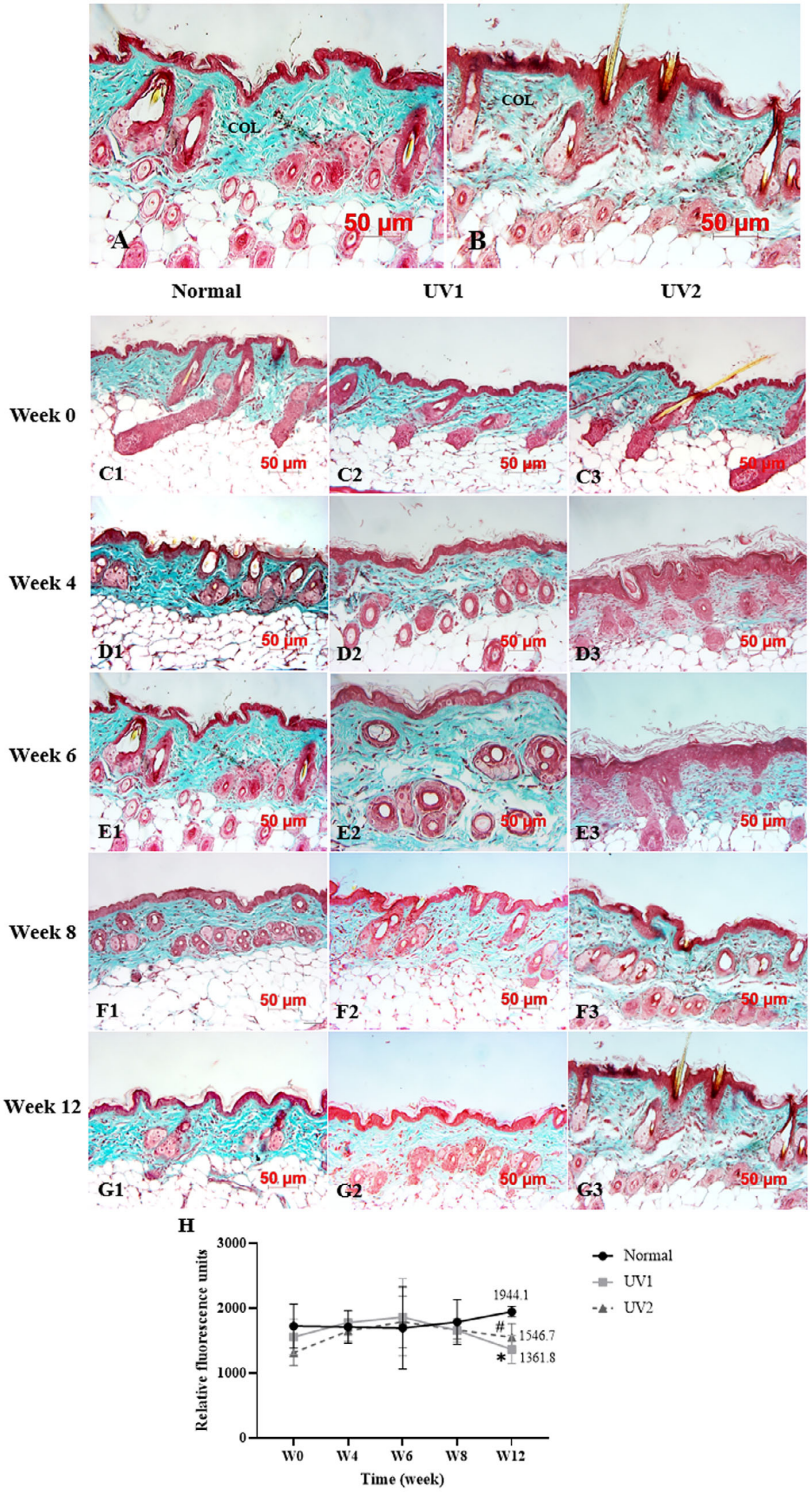
Histological alterations of collagen qualification using Masson's trichrome staining. Collagen (COL) density expression between normal mice (A) and UV‐induced aged mice (B). Histological alterations of collagen quantification of three mice groups at Week 0 (C1–C3), 4 (D1–D3), 6 (E1–E3), 8 (F1–F3), and 12 (G1–G3). Collagen intensity analyzed by Image J software. *n* = 3 per group; *p < 0.05, UV1 versus normal group; *p < 0.05, UV2 versus normal group. Data are presented as mean ± SD. One‐way ANOVA was used for the comparisons (H). ANOVA, analysis of variance; SD, standard deviation; UV, ultraviolet.

The collagen fiber quantity in the dermis was assessed using Masson's Trichrome staining. In normal skin samples, collagen fibers appeared bright blue, dense, and homogeneous (Figure 4A). In contrast, UV‐irradiated samples showed fragmented, disorganized collagen fibers‐stained light blue, interspersed with numerous blank spaces (Figure 4B).

At Week 0, collagen quantity was standard blue and homogeneous across all three groups normal, UV1, and UV2 (Figure 4C1, C2, and C3), and remained unchanged in the normal group by Weeks 4, 6, 8, and 12 (Figure 4D1, E1, F1, and G1). However, at Weeks 4, 6, 8, and 12, there was a noticeable reduction in bright blue‐stained material, replaced by light blue‐stained fibers and numerous white spaces in the UV1 (Figure 4D2, E2, F2, and G2) and UV2 (Figure 4D3, E3, F4, and G3) groups.

The intensity of collagen staining slightly increased in the UV groups at Weeks 0, 4, and 6 but gradually decreased by Weeks 8 and 12. By Week 12, the collagen intensity in the UV1 and UV2 groups was significantly lower than in the normal group, measuring 1361.833 ± 215.87 and 1546.70 ± 210.13, respectively, compared to 1944.13 ± 80.56 in the normal group (*p* < 0.05) (Figure 4H).

### 3.5 Effects of UV Irradiation on Collagen and MMPs Gene Expression

**FIGURE 5:**
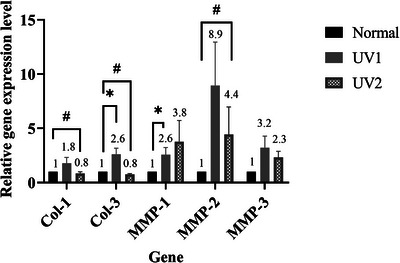
Gene expression of Collagen 1, Collagen 3, MMP‐1, MMP‐2, and MMP‐3 in skin tissue at Week 12. *p < 0.05, UV1 versus normal group; ^#^ *p* < 0.05, UV2 versus normal group. *n* = 6 per group. Data are presented as mean ± SEM. Mann–Whitney test was used for the comparisons. MMP, matrix metalloproteinase; SEM, standard error of mean; UV, ultraviolet.

By Week 12, mRNA levels of Collagen 1 and Collagen 3 increased in the UV1 group and slightly decreased in the UV2 group compared to the normal group. The differences were statistically significant between UV2 and normal group (*p* < 0.05). The mRNA expression levels of MMP‐1, MMP‐2, and MMP‐3 showed a marked elevation in the UV groups compared to the normal group, with a significant difference observed in MMP‐1 expression between the UV1 and normal groups (*p* < 0.05), and in MMP‐2 expression between UV2 and normal group (*p* < 0.05). Although MMP‐3 mRNA expression appeared higher in the UV1 and UV2 groups compared to the normal group, the differences were not statistically significant (*p* = 0.36 and *p* = 0.08, respectively) (Figure 5).

### 3.6 Evaluation of Aged‐Skin Recovery After 8 Week‐Finished Irradiation

**FIGURE 6:**
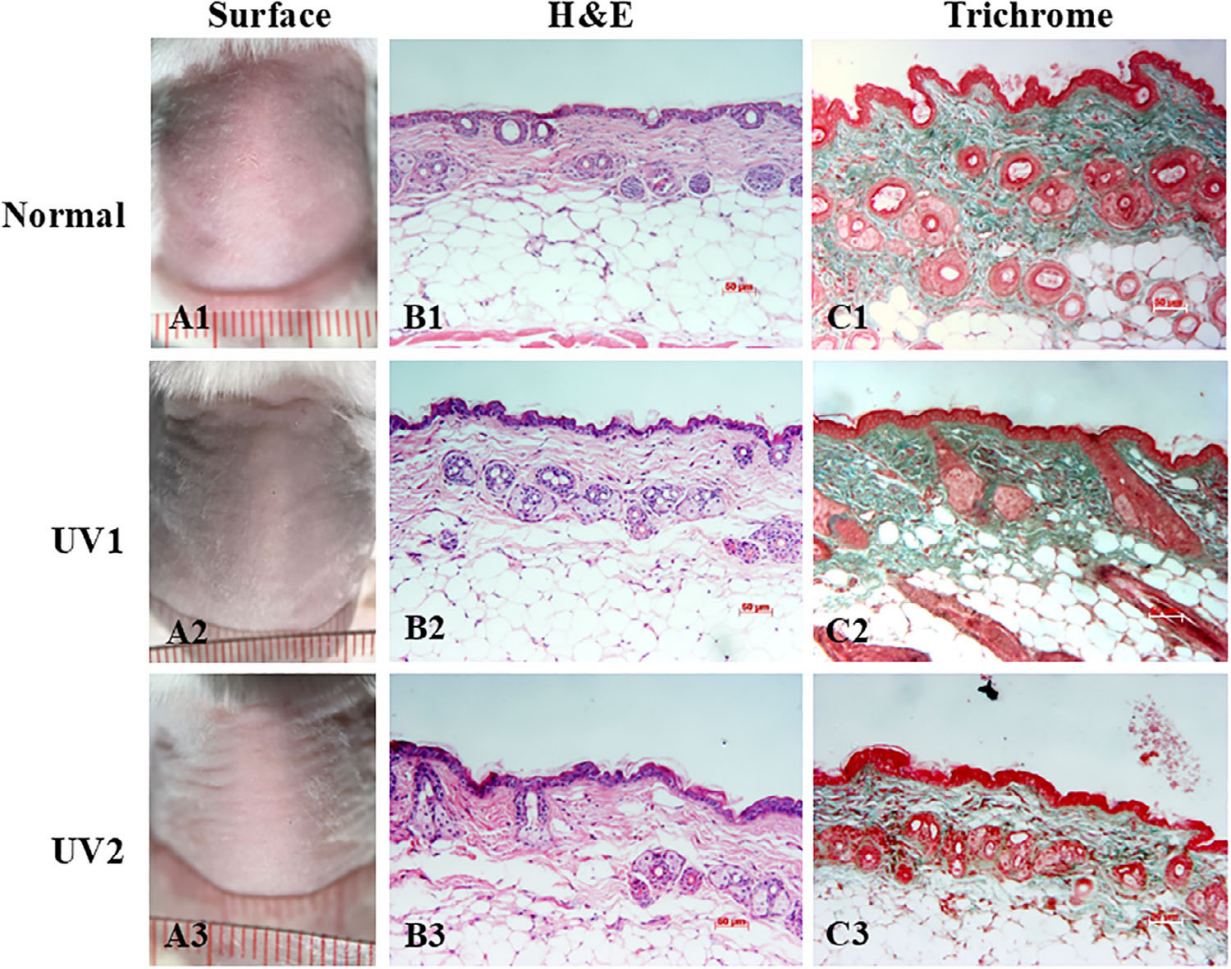
Aged‐skin recovery after 8 week‐finished irradiation. Aging signs of three mice groups assessed by wrinkles formation (A1–A3), epidermal thickness (B1–B3), and collagen density (C1–C3) changed after ultraviolet (UV) irradiation completion. Scale bar = 50 µm; magnification: ×20.

Eight weeks after completing UV irradiation, the surface of the skin appearance revealed persistent deep transverse wrinkles in both UV groups (Figure 6A2 and A3), compared to that in the normal group (Figure 6A1), with more pronounced wrinkles in the UV2 group (Figure 6A3).

Histological analysis using H&E staining indicated that the epidermal thickness nearly returned to normal in the UV1 group (Figure 6B2) but remained thickened in the UV2 group (Figure 6B3), compared to the normal group (Figure 6B1). Masson's Trichrome staining highlighted ongoing collagen degradation in the UV2 group, characterized by thin, sparse light blue‐green‐stained fibers and numerous blank spaces in the dermis (Figure 6C3), in contrast to the dense, homogeneous collagen bundles observed in the normal group (Figure 6C1). In the meanwhile, collagen bundles in the UV1 group showed less typical signs of aging (Figure 6C2). These findings demonstrate that skin tissues continued to exhibit signs of aging and showed no significant recovery even 8 weeks after the cessation of UV irradiation.

## 4 Discussion

This study aims to establish a BALB/c mouse model of photoaging by long‐term UV irradiation, with precise aging assessment. Skin‐aging mouse models can be established using various animal strains, including hairless mice, BALB/c mice, Lewis rats, Wistar rats [19], and Kunming mice [20].

The use of hairless mice in skin photoaging models is prevalent because of their inherent UV light susceptibility, eliminating the need for depilation that can cause inflammation [6]. However, hairless mice lack normal hair cycle development, potentially limiting their resemblance to human skin photoaging. The widely used SKH‐1 hairless mice face challenges due to their outbred status, genetic variation, and unsuitability for transplantation studies, making them less ideal for cutaneous actinic aging research [6]. Additionally, SKH mice are less susceptible to UVB irradiation and primarily exhibit edema with neutrophil infiltration post‐UVB exposure, suggesting they are more suitable for acute sunburn models rather than chronic photoaging models [21].

In contrast, inbred‐haired BALB/c mice offer genetic uniformity, affordability, and more natural skin features such as hair follicles, making them appropriate models for studying skin photoaging [7]. Mice with normal hair and intact follicular structures are valuable for providing insights into follicular aging, such as hair follicle miniaturization and the decline of hair follicle stem cells [22]. The presence of functional hair follicle structures in BALB/c mice influences the skin's response to UV radiation in the context of photoaging. Specifically, the hair follicle, particularly the bulge region, contains epidermal stem cells that are capable of regenerating the epidermis following injury and contribute to tissue repair and the maintenance of epidermal thickness. During aging, the number and functionality of these stem cells decline, resulting in diminished skin regeneration and repair capacity [22]. Although SKH‐1 mice possess hair follicles, these structures are degenerated and functionally inactive, thereby limiting the interpretation of findings obtained from this model to human skin. In addition, several studies have suggested that BALB/c mice may be a more suitable strain than SKH‐1 mice for investigating the immune response of the skin to chemical exposures. In particular, BALB/c mice exhibited increased neutrophil infiltration and upregulated expression of cytokines associated with inflammation and Th2 responses, whereas SKH‐1 hairless mice did not show significant alterations in neutrophil or dendritic cell populations [7]. These factors are also implicated in the physiological processes underlying skin aging. Therefore, we chose BALB/c mice for establishing an anti‐aging mouse model.

We also noticed that some previous studies did not develop precise and accurate criteria to evaluate photoaging signs, leading to misunderstandings and over‐extrapolation [23, 24]. By exposing BALB/c mice to extended periods of UV light, the study seeks to simulate chronic skin photoaging observed in humans. This approach intends to understand the mechanisms and manifestations of long‐term UV‐induced skin damage, mirroring chronic photoaging processes observed in human skin. The establishment of a BALB/c mouse photoaging model enables researchers to explore the molecular, histological, and clinical dimensions of chronic UV‐induced skin damage. Although BALB/c mice are an inbred strain with relatively consistent genetic backgrounds, inter‐individual variability in response to UV exposure may still occur. Subtle differences in skin thickness, baseline oxidative stress, or local immune responses can influence the extent of skin damage. In this study, efforts were made to minimize such variability by using age‐, weight‐, and sex‐matched mice, standardizing housing conditions, and applying consistent UV exposure protocols. Moreover, an appropriate sample size was determined using a validated formula to ensure sufficient power for the findings. Through these investigations, researchers aim to enhance the comprehension of the mechanisms underlying photoaging and to explore potential interventions or treatments for the detrimental effects of prolonged sun exposure on human skin.

Various UV irradiation protocols have been employed in research, with differences in UV dosage, duration, and wavelength. For example, Kim et al. exposed female albino mice to UVA irradiation at a total dose of 3 kJ/cm^2^ over 10 weeks, leading to flaccid, wrinkled skin [25]. This dose was higher than the UVA dose used in the current study (51.8 J/cm^2^). Another study by Ropke et al. irradiated albino nude mice with UVB at a cumulative dose of 6.16 J/cm^2^ over 22 weeks, resulting in furrow formation and thickening of the granular layer in the skin [26]. Sukwha Kim et al. reported a total UVB irradiation dose of 6.9 J/cm^2^ to cause photoaging in BALB/c nude mice, which is slightly higher than the UVB dose applied in our study (5.53 J/cm^2^) [27]. A recent study by Meifen Lin et al. used three different mouse species (C57BL/6J, ICR, and KM) to develop a chronic photoaging model with gradually increasing irradiation times over 40 days, from 10 min on Days 1–5, 20 min on Days 6–10, to 30 min on Days 11–40 [28]. Another study [21] compared visible changes after single UVB irradiation between three commonly used mouse strains, C57BL/6N, Balb/c, and SKH‐1 reported that dark hair‐possessing C57BL/6 mice are more sensitive to UVB irradiation than albino Balb/c and SKH‐1 mice. These studies highlight the diverse approaches to creating skin aging models using UV irradiation, each with its methods, dose regimens, and observed outcomes. Some studies employ high doses over short periods, while others utilize gradually increasing doses over longer duration, highlighting the versatility and complexity of experimental design in skin aging research [29, 30].

In this study, a combination of UVA and UVB light was used to simulate natural sunlight exposure and affect multiple skin layers, which contributes to better extrapolation. Besides, the coordination of UVA and UVB leads to lower UV energy utilized in the current study compared to other previous studies [26, 27]. Furthermore, this combination also helped to reduce the duration of UV irradiation, compared to some studies with sole UV type [26, 31]. In our study, two dosage levels (1 MED and 2 MED) were tested over 12 weeks to determine the optimal cumulative dose for chronic inflamed photoaging while avoiding acute photodamage like sunburn. 1 MED was defined as the lowest UV dose to produce visible erythema after 24 h without causing edema, similar to human MED tests. In our study, 1 MED equaled 540 mJ/cm^2^ UVA and 57.6 mJ/cm^2^ UVB, comparable to the 1 MED (54.3 mJ/cm^2^) after single UVB irradiation on BALB/c mice in Gyöngyösi et al.’s study [21]. Different studies have reported varying MED values for BALB/c mice, such as 200 and 150 mJ/cm^2^, which are higher than those used in the current study, with irradiation periods being shorter, typically around 6 weeks [27, 32]. However, the photoaging signs in those studies were not obvious.

In the current study, the results showed that there was an initial increase in body weight in the UV1 group, followed by a slight decrease at Week 8, and then exposed significant differences compared to the normal group at Week 12. This suggests a potential impact of UV1 dose on overall health of mouse that requires further investigation, while UV2 dose did not cause any significant alteration on the weight of mouse in the UV2 group compared to the normal group, indicating that the UV2 dose might be more applicable.

The assessment of skin aging characteristics, including wrinkles, skin hydration, elasticity, and thickness, provided more valuable criteria throughout the study period compared to other research on BALB/c mice [21, 32, 33]. UV light causes damage to the skin, affecting both the epidermis and dermis. The stratum corneum of the epidermis acts as a barrier preventing excessive loss of hydration from the skin. Therefore, damaged skin exhibits dryness and reduces moisture levels. To assess skin hydration, the dorsal skin of each mouse was examined using an appropriate probe of the Corneometer equipment. As depicted in the results, UV exposure in two doses decreased skin hydration in mice, with significant differences observed compared to normal mice at Weeks 6 and 12. UV irradiation leads to dehydration and loss of connective tissues in the dermis, resulting in skin laxity. Skin elasticity was assessed by measuring skin snapback time over 12 weeks using the pinch test. The results indicated that the skin snapback time of the UV‐exposed groups was longer than that of the normal group, with significant differences observed from Weeks 8 to 12. The UV2 group exhibited the highest snapback time compared to others at Week 12, demonstrating that the UV2 dose caused more damage to the skin.

Photoaged skin appears rough and thickened in surface morphology; therefore, skinfold thickness measurement using a digital caliper might provide a valuable indicator for determining actinic aging. Although increasing skin thickness in normal mice may be due to age‐related weight gain, thickened skin was more prominent in irradiated mice due to a skin feedback mechanism and dysfunction against photodamage, including epidermal hyperplasia and anormal large extracellular structure in the dermis. In this study, the effect of weight on skin thickness was controlled because there were no significant differences in weights between mouse groups from Week 0 to Week 8. At Week 12, the weight of mice in the UV1 group was slightly lower than the normal group. Skinfold thickness was significantly greater in the UV‐exposed groups compared to the normal group at Weeks 8 and 12.

Morphological changes observed including the development of deep wrinkles, furrows, laxity, and dryness. Utilizing digital microscopy, the aged skin of mice appeared pale, dry, rough, and flaccid, with deep horizontal wrinkles perpendicular to the central vertical axis of the back. In contrast, normal mice typically exhibited fine vertical wrinkles along the sides of the back. These photoaged mouse skin features were consistent with previous studies [15]. Initially, these wrinkles appeared on the upper back skin, gradually extending to cover the full back by Weeks 8 and 12. Notably, mice in the UV2 group exhibited more pronounced skin aging characteristics compared to those in the UV1 group.

Moreover, UV irradiation led to a significant increase in Bissett wrinkle scores from Week 4. This scoring system, which assesses the severity of wrinkles, demonstrated a progressive worsening of skin aging features over the course of the study period. Although most skin aging characteristics were clearly detected at Week 8 and maintained through Week 12, deep and fully developed skin wrinkles were only clearly observed at Week 12, making this time point ideal for further treatment investigation. These findings underscore the effectiveness of UV irradiation in our study to induce skin aging phenotypes in the mouse model and highlight the importance of assessing wrinkle formation as a key aspect of photoaging research.

Histologically, epidermal thickening and dermal elastosis are key indicators of photoaging [34, 35]. Skin histology with H&E stain demonstrated both epidermal and dermal thickening, and the accumulation of elastin fibers in the dermis, while Masson's Trichrome stain revealed the destruction of collagen fibers. Our study showed a gradual thickening of both the epidermis and dermis following UV exposure in H&E stain, which is consistent with previous research [27]. Epidermal thickening can be attributed to the initial response of keratinocytes to UV irradiation via the activation of epidermal growth factor receptor (EGFR), since EGFR activation is mitogenic in many cell types including keratinocytes and suppresses apoptosis, leading to transient keratinocyte proliferation [36]. On the other hand, level of PCNA expression, severed as a biomarker of proliferation, increased dramatically after irradiation, as well as decreased differentiated cells coincided with increase in epidermal hyperplasia [37]. However, prolonged exposure of keratinocytes to UV light can cause irreversible DNA damage, eventually resulting in apoptosis and a reduction in epidermal thickness [38]. In our study, the epidermal thickness was consistently higher in the UV‐exposed groups compared to the normal group from Week 4 to Week 8, after which it gradually decreased from Week 8 to Week 12, although it was more pronounced compared to normal group. This result aligns with findings from several previous studies [21, 27] and resembles the characteristics of aging in humans. It is important to note that the manifestation of skin aging in humans is influenced by skin phototype. Specifically, individuals with Phototypes I–II are more prone to epidermal atrophy, whereas those with Phototypes III–IV tend to exhibit epidermal thickening [39]. Additionally, intrinsic (chronological) aging is associated with epidermal thinning, primarily due to decreased cell turnover and progressive tissue atrophy [40].

Our results also demonstrated an increase in dermal thickness in the UV‐exposed groups compared to the normal group, likely due to the development of dermal cysts, elastosis, and the replacement of collagen fibers by glycosaminoglycan in the dermis [30]. In H&E‐stained samples from the normal group, collagen bundles appeared pink. However, in UV‐exposed groups, collagen fibers were broken, tangled, disorganized, and fragmented, being replaced by dense, abnormally basophilic‐stained elastin fibers. This phenomenon, known as elastosis, typically results in a thickened dermis [30]. Dermal elastosis was observed in our study at Week 12, consistent with the findings of Bissett's research, where elastosis was observed only after prolonged exposure of 16 weeks [15]. These features closely resemble the signs of actinic aging observed in humans.

Furthermore, to deeply investigate collagen degradation using Masson's Trichrome staining, collagen appeared dark blue in normal skin and became light blue with spaces in photoaged skin. Our study indicated an early increase in collagen quantity due to UV irradiation, followed by a decrease in collagen amount with prolonged exposure. Previous reviews have suggested that UV light initially stimulates collagen synthesis but subsequently reduces collagen synthesis rather than directly destroying collagen after chronic exposure [30]. Collagen intensity, measured by ImageJ software, also demonstrated that the intensity in the UV‐exposed groups was significantly lower than in the normal group at Week 12.

To investigate the effect of UV‐AB on collagen and metalloproteinase (MMP) expression in skin tissue, we analyzed the expression of wrinkle‐associated genes, including *COL1A1*, *COL3A1*, collagenase (*MMP1*), gelatinases (*MMP2*), and stromelysin (*MMP3*). The gene expression analysis revealed a biphasic response of collagen synthesis to UV exposure. At the UV1 (lower UV) dose, *COL1A1* and *COL3A1* mRNA expression increased, likely representing an early reparative response to extracellular matrix damage. In contrast, UV2 (higher UV) exposure resulted in a marked suppression of collagen transcription, consistent with cumulative cellular injury and senescence‐related signaling. These results are consistent with previous studies reporting that low‐dose or short‐term UV exposure can transiently stimulate collagen gene expression as part of an initial repair response, whereas prolonged or higher UV exposure leads to inhibition of collagen synthesis and enhanced matrix degradation [3, 4, 30].

Moreover, the predominant increase was observed in collagen Type III, which typically increases during the early wound‐healing phase [41], supporting the notion that this represents an early transcriptional attempt to repair UV‐induced damage. Consistently, a previous study also reported elevated Type III collagen level after 12 weeks of low‐dose UVB irradiation (five times per week at 1/2 MED per exposure) [30].

Although collagen mRNA expression was elevated, histological analysis revealed a reduction in collagen content in the UV1 group. Several complementary mechanisms can explain this apparent discrepancy between increased *COL* mRNA and decreased collagen deposition. First, mRNA expression represents an early and transient response to tissue injury, reflecting a compensatory attempt by fibroblasts to repair the extracellular matrix. However, this transcriptional upregulation may not translate into effective collagen restoration due to concurrent activation of matrix‐degrading enzymes and limitations in translation and post‐translational processing. The synthesis and maturation of fibrillar collagen require multiple sequential steps (translation, procollagen processing, cross‐linking, and extracellular deposition) that occur over a longer timescale [41]. Second, UV exposure is known to strongly induce MMPs and other proteolytic enzymes [4], as observed in the UV1 group; hence, newly synthesized collagen may be rapidly degraded, leading to an overall reduction in histologically detectable collagen fibers despite elevated *COL* mRNA levels.

In the UV2‐exposed group, the expression of Collagen 1 and Collagen 3 genes slightly decreased, while the expression of matrix‐degrading metalloproteinases such as MMP‐1, MMP‐2, and MMP‐3 genes increased. These findings align with previous studies, suggesting that fibroblast senescence and a loss of mechanical stimulation in aged tissues may reduce collagen synthesis. Additionally, UV irradiation increases the levels and activity of MMPs, leading to the degradation and fragmentation of collagen and elastin fibers in the dermis through AP‐1 induction [42, 43]. Taken together, these results demonstrate that the UV2 dose appears to be optimal for establishing a UV‐induced skin photoaging model.

Further investigation showed that the damaged skin remained aged based on surface morphology and histology examination after an 8‐week post‐irradiation period, suggesting this time point may be suitable for intervention experiments. One previous study also reported no regression in skin aging features over 15 weeks post‐UV irradiation [15]. Nevertheless, additional criteria are needed to detect the long‐term consequences of the UV‐induced model beyond morphology and pathology. To gain a deeper understanding of the photoaging mechanism, further research should involve larger sample sizes and more techniques to investigate alterations in gene and protein expression related to the aging process, as well as the roles of other predominant factors such as oxidative stress and the microenvironment.

Although various photoaged skin rodent models have been developed, the current study emphasized that chronic UV exposure in BALB/c mice effectively produces aged characteristics closely resembling human skin. Along with other aging features, the hallmark and most challenging formation marker for photoaging, solar elastosis, can be successfully created within 12 weeks, making this time point suitable for developing a photoaging model. Additionally, using a low‐fluence UVA and UVB coordination source helps to mimic solar light radiation, which can simulate skin damage symptoms resemble to those observed in human skin. Moreover, few studies have evaluated both qualitative and quantitative criteria with reliable analysis methods, such as measuring skin hydration with validated equipment or assessing collagen density using the ImageJ software with the color deconvolution plugin for analysis. This approach provides valuable fundamentals for evaluating photoaging features.

Our study would be strengthened by including additional information such as the success rate of the model based on clinical or histological criteria, as well as the incidence of unexpected findings. However, we note that no severe adverse effects, including skin edema, blistering, or ulceration, were observed in any of the animals. Furthermore, several aspects could be addressed to enhance the robustness of future research. These include controlling inter‐individual variability, increasing the sample size, and the number of experimental replicates to improve statistical power. Additionally, further assessments of skin aging histological characteristics may be considered, such as specific elastin and extracellular matrix staining, evaluation of inflammatory cytokines and reactive oxygen species (ROS) generation, as well as identifying senescent fibroblasts within the tissue. These refinements would contribute to a more comprehensive and reliable experimental model.

## 5 Conclusion

Our study successfully validates the BALB/c mouse as an effective model for simulating and studying photoaging by concurrently emitting UVA and UVB light with total dosage equivalent to 51.8 J/cm^2^ UVA and 5.53 J/cm^2^ UVB in 12 weeks. Significant UV‐induced skin aging markers, such as increased skin thickness, wrinkle formation, reduced hydration, and decreased elasticity, were observed. Histological and molecular analyses revealed key changes including epidermal hyperplasia, dermal thickening, collagen degradation, and altered expression of MMPs and collagen. These findings not only demonstrate the relevance of the model to human photoaging but also pave the way for future research into anti‐aging therapies and photoprotection strategies. The BALB/c mouse model offers a promising platform for advancing our understanding of photoaging mechanisms and for testing interventions aimed at mitigating the detrimental effects of UV exposure on the skin.

## Funding

This research is funded by VNUHCM‐US Stem Cell Institute, University of Science, Viet Nam National University Ho Chi Minh City, Ho Chi Minh City, Viet Nam (Grant number: 05/2023/SCI‐KHCN).

## Ethics Statement

The use of animals in the current study was approved by the Institutional Animal Care and Use Committee, VNUHCM‐US Stem Cell Institute (approval no. 210103/SCI‐AEC, date 20/01/2021).

## Conflicts of Interest

The authors declare no conflicts of interest.

## Supporting information



Supporting Information: “srt70353‐sup‐0001‐FigureS1.Png”

## Data Availability

All data generated or analyzed to support the findings of this study are available from the corresponding author upon request.
