# Supplementary figures and images for: Establishment of a BALB/c Mouse Model for Photoaged Skin: Insights into UV‐Induced Dermatological Changes
Source: Skin Res Technol. 2026 Apr 23;32(4):e70353. doi: 10.1111/srt.70353 (PMC13103728; doi:10.1111/srt.70353)

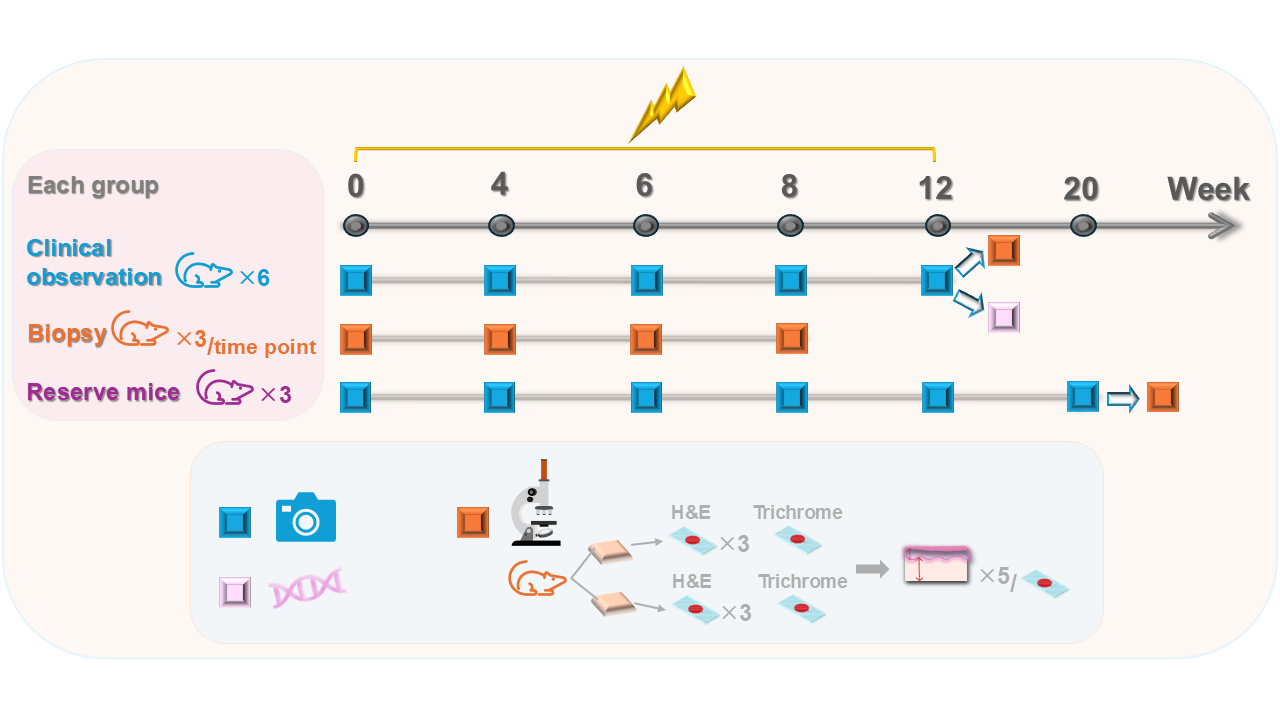

Supplement: Supplementary file 1 — Supporting Information: “srt70353‐sup‐0001‐FigureS1.Png” [file SRT-32-e70353-s001.png]
